# A novel frameshift *TBX4* variant in a family with ischio-coxo-podo-patellar syndrome and variable severity

**DOI:** 10.1007/s13258-024-01589-5

**Published:** 2024-10-28

**Authors:** Giada Moresco, Ornella Rondinone, Alessia Mauri, Rita Gorgoglione, Daniela Maria Grazia Graziani, Michal Dziuback, Monica Rosa Miozzo, Silvia Maria Sirchia, Luca Pietrogrande, Angela Peron, Laura Fontana

**Affiliations:** 1 Medical Genetics, Department of Health Sciences, Università degli Studi di Milano, Milan, Italy; 2 Research Laboratories Coordination Unit, Fondazione IRCCS Ca Granda Ospedale Maggiore Policlinico, Milan, Italy; 3 Medical Genetics, ASST Santi Paolo e Carlo, Milan, Italy; 4 Orthopedics and Traumatology Unit, ASST Santi Paolo e Carlo, Milan, Italy; 5 Orthopedics and Traumatology, Department of Health Sciences, Università degli Studi di Milano, Milan, Italy; 6 Present Address: Department of Biomedical and Clinical Sciences, Pediatric Clinical Research Center “Romeo ed Enrica Invernizzi”, University of Milan, Milan, Italy; 7 Present Address: Medical Genetics Laboratory, Fondazione IRCCS Ca’ Granda Ospedale Maggiore Policlinico, Milan, Italy; 8 Division of Medical Genetics, Meyer Children’s Hospital IRCCS, Florence, Italy; 9 Present Address: Department of Experimental and Clinical Biomedical Sciences “Mario Serio”, Università degli Studi di Firenze, Florence, Italy

**Keywords:** Exome sequencing, TBX4, Congenital patellar dislocation, Variable intrafamilial severity

## Abstract

**Background:**

Congenital anomalies of the knee are a spectrum of rare disorders with wide clinical and genetic variability, which are mainly due to the complex processes underlying knee development. Despite progresses in understanding pathomechanisms and associated genes, many patients remain undiagnosed.

**Objective:**

To uncover the genetic bases of a congenital patellar dislocation affecting multiple family members with variable severity.

**Methods:**

We performed ES in the proband and his father, both showing bilateral patellar dislocation, his sister with a milder similar condition, and his unaffected mother. Sanger sequencing was then performed in the proband’s brother and paternal aunt, both affected as well.

**Results:**

ES and Sanger sequencing identified the presence of the novel heterozygous frameshift mutation c.735delT in the *TBX4* gene in all affected family members. *TBX4* is associated with autosomal dominant ischio-coxo-podo-patellar syndrome with/without pulmonary arterial hypertension (ICPPS, #147891), reaching a diagnosis in the family. Intrafamilial clinical heterogeneity suggests that other factors might be involved, such as additional variants in *TBX4* or in other modifier genes. Interestingly, we identified three additional variants in the *TBX4* gene in the proband only, whose phenotype is more severe. Despite being classified as benign, one of these variants is predicted to disrupt a splicing protein binding site, and may therefore affect *TBX4* alternative splicing, accounting for the more severe phenotype of the proband.

**Conclusion:**

We expand and further delineate the genotypic and phenotypic spectrum of ICPPS. Further studies are necessary to shed light on the potential effect of this variant and on the variable phenotypic expressivity of *TBX4*-related phenotypes.

**Supplementary Information:**

The online version contains supplementary material available at 10.1007/s13258-024-01589-5.

## Introduction

Congenital anomalies of the knee are a spectrum of rare disorders with wide clinical and genetic variability and an estimated incidence of about 1 in 10,000 live births (Bongers et al. 2005). Among them, congenital patellar dislocation (CPD; OMIM %169000) is one of the most severe forms and should be suspected in every patient presenting at birth with knee valgus, flexion contractions, and external rotation of the tibia, which evolves into a severe phenotype compromising lower limb stability and ambulation (İlyas et al. 2018).

The complex processes underlying the development of the patella in humans begin during embryogenesis with the first patellar anlage appearing as early as 7 gestational weeks and developing into a cartilage mass. Patellar ossification begins at 5–6 years of age and is completed during adolescence (Samuels and Campeau 2019). The genetics of the patella reflects the complexity of this developmental process, and genes associated with patellar defects, such as abnormal, reduced, or absent bony patellae (hypoplasia or aplasia), can be divided into three categories: genes involved in (i) limb specification and joint pattern formation; (ii) DNA replication and chromatin structure; and (iii) bone development and differentiation (Samuels and Campeau 2019). Most of these disorders exhibit an autosomal dominant inheritance pattern. When biallelic pathogenic variants are present, patients are usually compound heterozygotes with at least one of the two mutations being a missense, because the complete loss of function of both alleles is usually lethal or causes extremely severe phenotypes (extensively reviewed in Bongers et al. 2005; Samuels and Campeau 2019; Vanlerberghe et al. 2018; Warman et al. 2011).

Ischio-coxo-podo-patellar syndrome with or without pulmonary arterial hypertension (ICPPS; OMIM #147891) is a rare autosomal dominant disorder with multiple skeletal abnormalities, including patellar aplasia or hypoplasia, described in less than 100 patients. Pelvic anomalies include bilateral absent or delayed ossification of the ischiopubic junction and infra-acetabular ax cut notches. Other major signs are a wide gap between the first and second toes (sandal gap), short fourth and fifth toes, and pes planus (Bongers et al. 2004). Pediatric-onset pulmonary arterial hypertension (PAH) is occasionally seen in association with ICPPS, without clear genotype-phenotype correlations (Kerstjens-Frederikse et al. 2013; Levy et al. 2016; Maddaloni et al. 2024). ICPPS is caused by heterozygous pathogenic variants in the *TBX4* gene (Bongers et al. 2004), involved in limb specification and joint pattern formation. Biallelic mutations in the *TBX4* gene are responsible for posterior amelia with pelvic and pulmonary hypoplasia syndrome (PAPPAS, #601360), a lethal condition. This gene encodes for the TBX4 protein (T-box transcription factor 4), a member of the evolutionarily conserved family of T-box–containing transcription factors, which functions as a key regulator of lung branching morphogenesis and hindlimb formation (Karolak et al. 2023).

Heterozygous *TBX4* variants are responsible for several conditions affecting both the respiratory and the skeletal systems, with severity ranging from mild to lethal. Indeed, patients show great heterogeneity with regard to both the clinical manifestations and the age of onset, suggesting the possible contribution of other genetic and non-genetic factors (Karolak et al. 2023). Co-inheritance of additional coding and noncoding variants, both in the *TBX4* locus and in other possibly correlated genes, has been proposed to explain differences in disease severity (Karolak et al. 2019, 2020), but complete understanding of the variable phenotypic expressivity represents one of the most challenging issues related to *TBX4* genetics.

In the present study, we performed exome sequencing (ES) to define the molecular diagnosis in a family affected by a form CPD with a suspected genetic etiology and an autosomal dominant inheritance pattern. This analysis allowed the identification of a novel frameshift mutation in the *TBX4* gene, leading to the diagnosis of ICCPS. Not only this discovery could facilitate a more precise clinical management of the patient, but also contributed to the expansion of known *TBX4* pathogenic variants. With the aim of explaining the variable intrafamilial severity in our family, we also checked the presence of additional *TBX4* variants, and we explored the hypothesis of a digenic inheritance or the contribution of other genes in defining the disease severity.

## Materials and methods

All family members included in the study provided written informed consent and authorization for the publication of clinical data and photographs.

### Case presentation

**Fig. 1:**
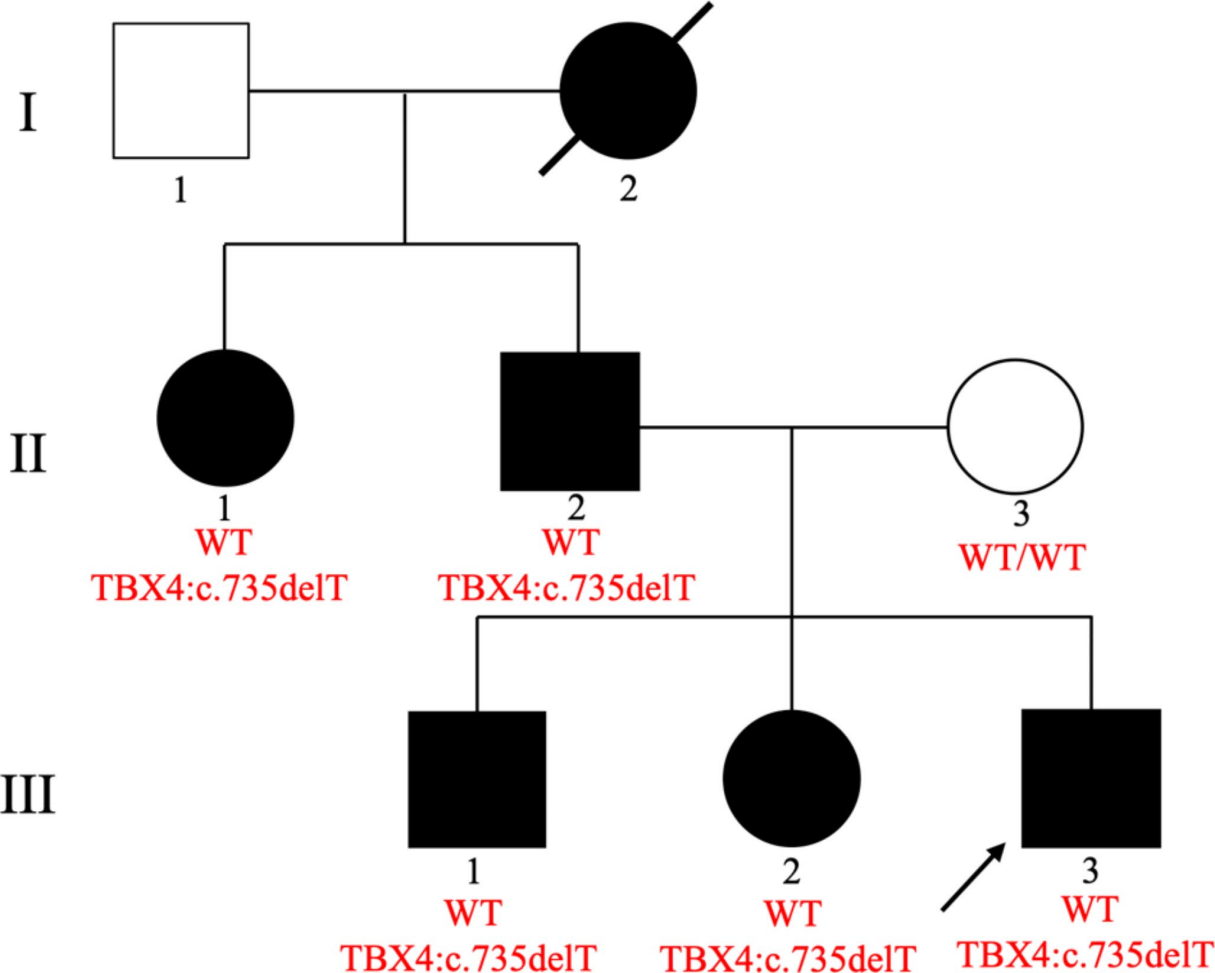
Pedigree of the family and genotype of *TBX4* for each tested family member

The family pedigree is reported in Fig. 1.

**Fig. 2:**
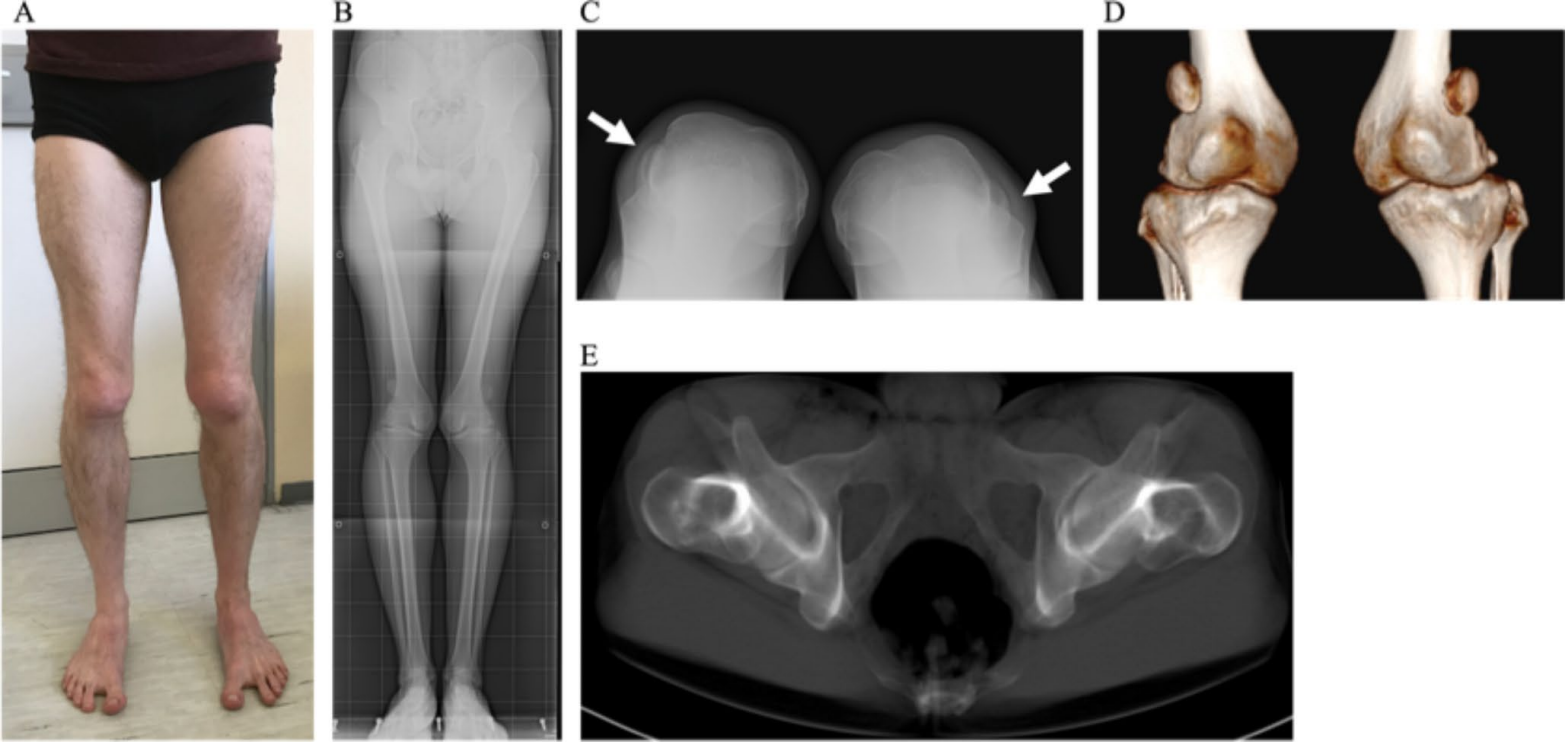
Clinical picture of the proband before surgery. **A** clinical aspect;** B** X-ray images in standing position that show internal torsion of the knees, with femoral valgus and tibial varus and dislocated patella; **C** sunrise view X-ray showing complete lateral dislocation of the hypoplastic patella and flat femoral trochlea on both sides (arrows); **D** 3D CT scan image of both dislocated patellae; (E) the retroversion of femoral neck seen in CT scan reconstruction

The proband (third born of non-consanguineous parents; III-3) is a 22-year-old man presenting with congenital patellar dysplasia, bilaterally dislocated, hypoplastic patella alta, iliac crest and femoral head misalignment, and sandal gap. He underwent surgery for Achilles tendon lengthening to solve left pes planus in childhood, and surgery for the correction of congenital dysplasia in the left knee at the age of 20 and in the right knee at the age of 22 years. The dislocated patella caused a relevant internal rotation of the distal femur, compensated by a severe anteversion of the femoral neck, identified on an axial study with a CT scan of the femur. Figure 2 reports the clinical picture (Fig. 2A) and X-rays (Fig. 2B and C) and CT scan (Fig. 2D and E) images before surgery.

**Fig. 3:**
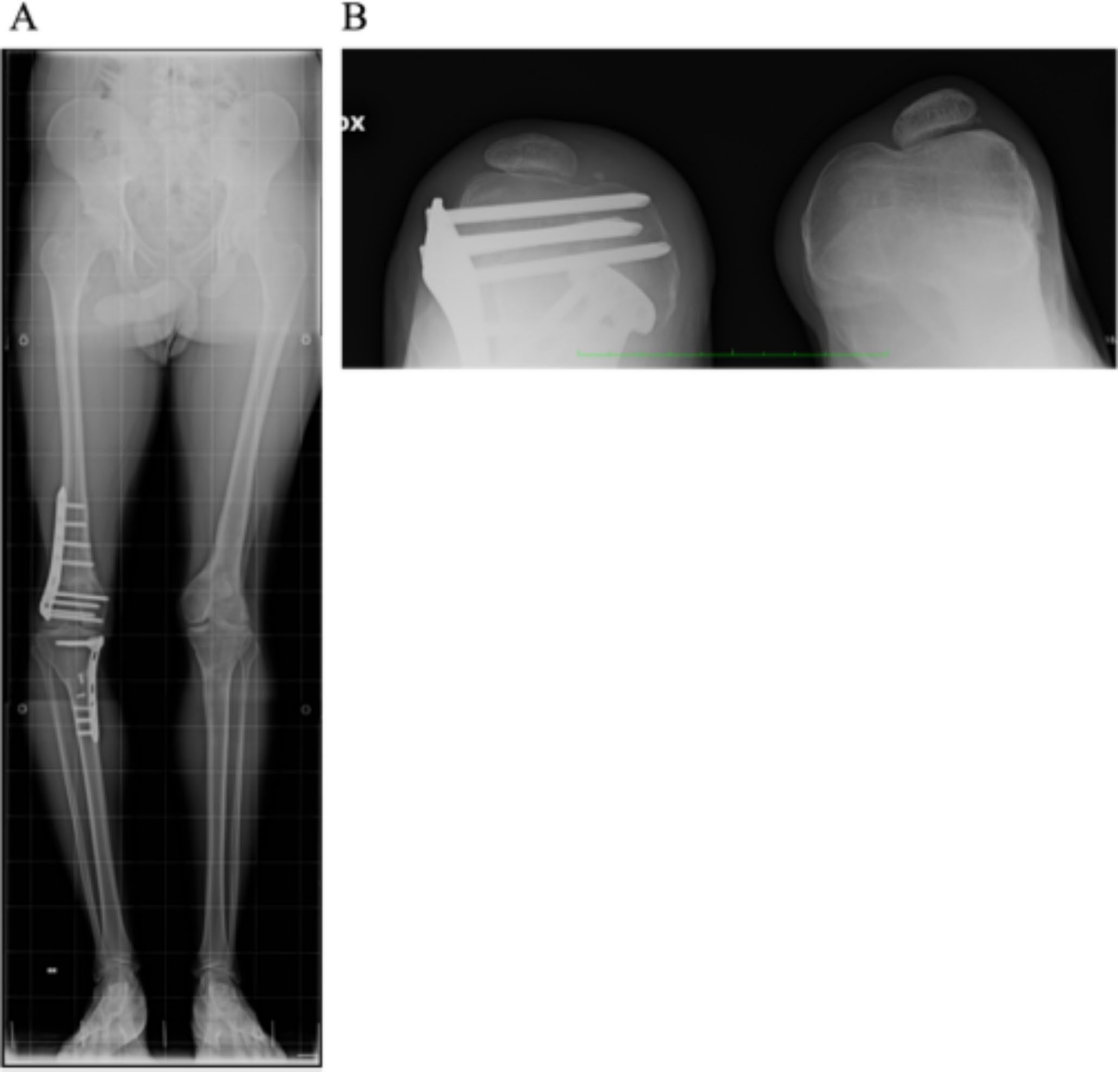
X-ray images of the proband after the second surgery, with hardware removed on the left side. **A** X-ray images in standing position and** B** sunrise view X-ray showing the hypoplastic patella now centered in the flat femoral trochlea on both sides

The surgical correction involved a one-stage double osteotomy, with a reductive varus osteotomy performed at the distal femur and an additive valgus osteotomy performed at the proximal tibia, utilizing the bone fragment obtained from the femur. At the same time, the tibial tubercle with the distal insertion of the severe hypoplastic patellar tendon was transposed distally and medially. After surgery, the proband rapidly gained strength in the knee, and the tendon became stronger and thicker within a few months. Although there was not a symmetrical correction of the varus-valgus in both sides, the functional result (shown in Fig. 3) was optimal.

The proband’s father (II-2) exhibits an overlapping phenotype, for which he underwent numerous surgeries in early adulthood with an unfavorable evolution, probably due to concomitance of a primitive immunodeficiency due to lack of immunoglobulins. The proband’s paternal grandmother (I-2; deceased) and aunt (II-1) also presented orthopedic issues. In particular, the paternal aunt reports surgical interventions following sports injuries (skiing and skating), and presents a lateralization of the patella, with lateral hyper-pression and flat femoral trochlea. The proband’s older brother (III-1) exhibits a phenotype similar to that of the proband, with trochlear dysplasia and a bipartite, laterally dislocated patella alta. Their sister (III-2) is affected as well, although manifesting a milder phenotype: she reports a previous reinsertion of the alar ligament of the rotula after the lateral dislocation following a sports trauma (skiing). With regard to the phenotype severity, the affected females exhibited a milder phenotype than the affected males, limited to minor patellar lateral dislocation that did not interfere with normal daily activities, although causing progressive degeneration of the cartilage.

No other relevant medical issues are reported in this family, and in particular, no pulmonary issues have been documented in childhood and thus far.

### ES and bioinformatic analysis

Genomic DNA was extracted from peripheral blood lymphocytes (PBLs) of the proband, his sister and his parents using the QIAsymphony DSP DNA Midi kit (QIAGEN, Hilden, Germany), according to the manufacturer’s instructions. Next Generation Sequencing (NGS) analyses were performed using the SureSelectQXT Clinical Research Exome V2 kit (Agilent, Santa Clara, CA) and libraries were sequenced by 151 bp paired-end reads on the Illumina NextSeq 550 platform (Illumina, San Diego, CA). Sequences were aligned through BWA-MEM v0.7.7 on GRCh37/hg19 and Picard v1.79, and variants were called through the GATK HaplotypeCaller v1.6 (Poplin et al. 2018). VCF files were then annotated and analyzed using the eVai software (https://www.engenome.com) and interpreted according to the American College of Medical Genetics and Genomics (ACMG) guidelines (Richards et al. 2015), using Varsome (Kopanos et al. 2019), Franklin by Genoox (https://franklin.genoox.com, 2022), TargetScan (Agarwal et al. 2015), disease-variant/gene databases (e.g., HGMD (Stenson et al. 2003), ClinVar (Landrum et al. 2018), OMIM (https://omim.org/, 2022), and a critical revision of the literature.

The identified variant was confirmed by Sanger sequencing (primer sequence and further details are available upon request).

Due to the unavailability of RNA and protein samples, functional studies have not been performed to investigate the functional consequences of the identified variant. We used the MutationTaster tool (Schwarz et al. 2014) to define the position of premature termination codons (PTCs) compared to the canonical ones and to predict the effect of nonsense-mediated decay (NMD) according to the rules governing this process (Lindeboom et al. 2016).

Digenic inheritance analysis in the proband (III-3) was performed by the DIVA software (https://www.engenome.com), with the “single proband mode”; information on the inheritance pattern of variants in the digenic combination was then deduced from trio-ES analysis. Predicted digenic mechanism is reported as TD/CO (True Digenic or Composite) or DM (Dual Molecular Diagnosis).

The TD mechanism (also known as Pure Digenic) emerges when both causative genes carry mutations in the patient’s genome, the CO mechanism (also known as Modifier) refers to variants that can influence disease outcomes, and the DM mechanism entails the co-occurrence of two distinct disorders, each caused by pathogenic variants in different genes (De Paoli et al. 2023).

## Results

**Fig. 4:**
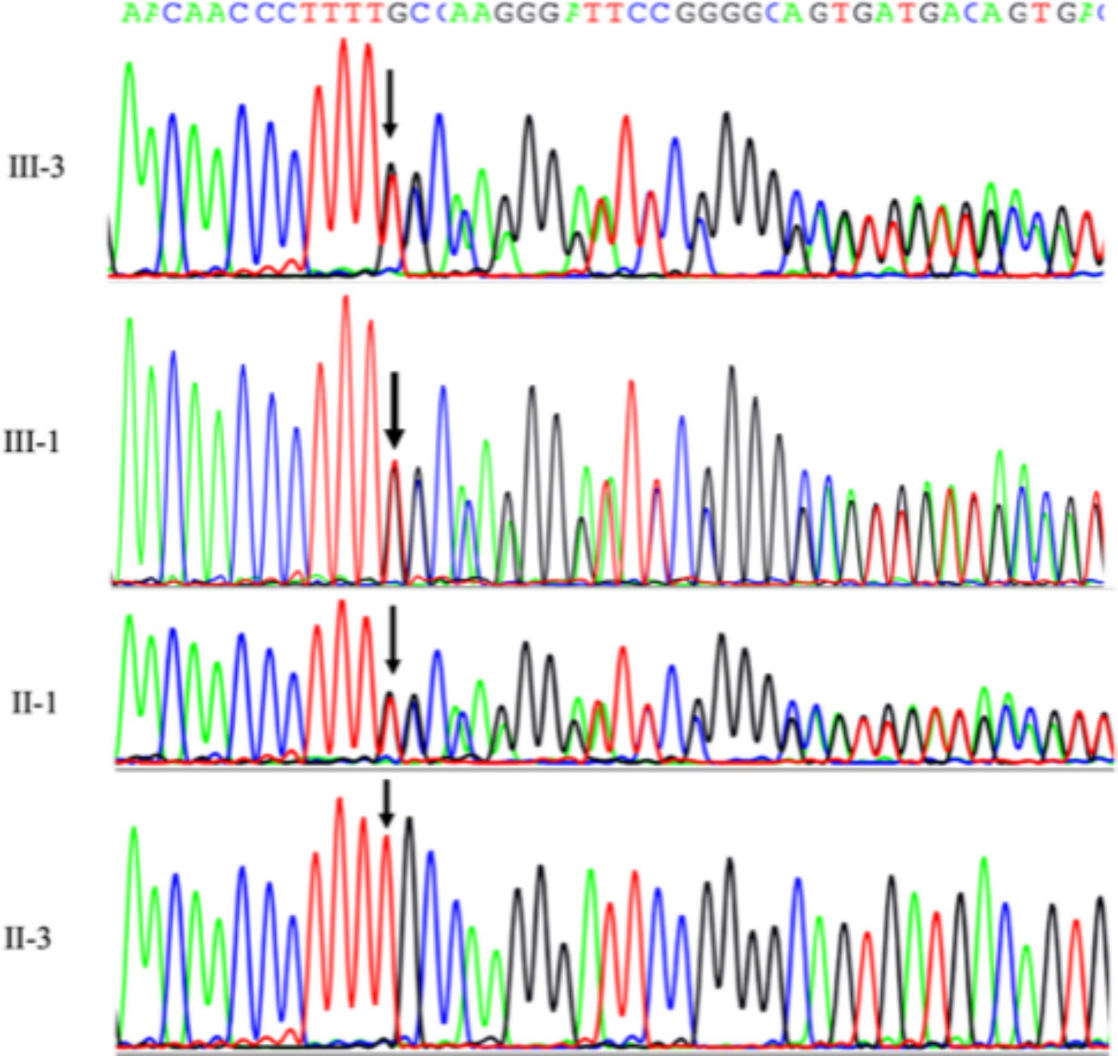
Sanger electropherogram of the *TBX4* mutation of the proband (III-3), his brother (III-1), the paternal aunt (II-1), and the wild-type unaffected mother (II-3)

An average of about 100,000 variants emerged from each ES, with a mean Q30 sequencing score of 83% and a uniform coverage above 100X. Variant prioritization allowed the identification of the novel frameshift variant c.735delT (p.Phe245Leufs*25) in exon 7 of the *TBX4* gene (NM_001321120.2) in all analyzed affected family members (II-2, III-2, III-3). Sanger sequencing confirmed the presence of the identified variant in the proband and its segregation in the family, including the elder brother and the paternal aunt (Fig. 4).

The c.735delT variant is localized at the end of the T-box DNA binding domain, and it is predicted by MutationTaster to cause a frameshift of 25 amino acids, after which a premature stop codon (PTC) is introduced, thus leading either to the generation of a truncated protein, lacking 277 amino acids out of a total of 546, or to NMD, according to the PTCs model (Lindeboom et al. 2016).

We then screened the *TBX4* locus for additional variants in both the coding and the noncoding regions captured by the probes included in the ES. Variants emerging from this further analysis are reported in Table 1. We identified six additional variants, of which one (c.276T>G) is shared by all affected family members, three are present only in the proband (c.941C>T, c.1227C>T, c.*707G>T), one (c.402-8G>A) was identified only in the proband’s sister (III-2) and the last one (c.17G>C) was found only in the proband’s father (II-2). Of these, three (c.276T>G, c.941C>T, c.1227C>T) are synonymous, one (c.17G>C) is a missense variant, one (c.402-8G>A) is a splicing/intronic variant, and the last one (c.*707G>T) is a 3’UTR variant, which is not predicted by the TargetScan in silico tool to impact on miRNA binding. Although all these variants are present in the general population with a frequency (MAF) above 5%, and thus classified as benign according to the ACMG criteria, we are not able to exclude their possible contribution to the variable phenotypic severity.

**Table 1:** Additional *TBX4* variants emerged from ES analysis, with respective ACMG classification. For each variant, an X represents the individual in whom it has been identified

TBX4 variants			Family member				
HGVS_Coding	HGVS_Protein	ACMG Classification	III-3 (proband)		III-2 (sister)	II-2 (father)	II-3 (mother)
c.735delT	p.Phe245fs	P (PVS1, PM2, PP4, PP1)	X		X	X	
c.276T>G	p.Ala92Ala	B (BA1, BS2, BP7, BP6)	X		X	X	
c.941C>T	p.Ala314Val	B (BA1, BS2, BP6)	X				X
c.1227C>T	p.Asp409Asp	B (BA1, BS2, BP6, BP7)	X				X
c.*707G>T	-	B (BA1, BS2, BP6, BP7)	X				X
c.402-8G>A	-	B (BA1, BS2, BP4, BP6)			X		
c.17G>C	p.Gly6Ala	B (BA1, BS2, BP6)				X	

*P* pathogenetic, *B* = benign

Moreover, the DIVA software (EnGenome) was interrogated to explore the possibility of either a digenic condition or a dual molecular diagnosis with the aim of explaining the different severity of the phenotype observed in the proband (III-3) and his father (II-2) compared to his sister (III-2). From this analysis 42 different possible combinations emerged (Table S1), including both digenic conditions and a dual molecular diagnosis involving the *TBX4* gene. However, no additional candidate gene correlated with the phenotype of the affected family members emerged from this analysis.

## Discussion

We performed ES in a family affected by an autosomal dominant form of congenital patellar dislocation, and we identified the novel frameshift variant c.735delT in the *TBX4* gene in all affected family members (II-2, III-2, III-3) and confirmed its presence by Sanger sequencing in the proband’s brother (III-1) and paternal aunt (II-1). This gene is responsible for ICCPS, thus allowing us to reach a conclusive diagnosis in this family.

The affected family members reported in this study show variable clinical severity: the males exhibit a more severe condition compared to the females (II-1 and III-2), who are mildly affected and able to perform sports, such as skiing. Although *TBX4* variants in ICPPS usually show high penetrance, this gene is also known to be characterized by variable expressivity and incomplete penetrance, especially in PAH families with segregating *TBX4* variants, thus suggesting the involvement of other environmental or genetic factors (Karolak et al. 2023). Kruse et al. (2008) hypothesized that the male predominance of idiopathic clubfoot caused by a microduplication involving *TBX4* could be explained by the Carter effect, which supports a multifactorial threshold model of inheritance (Kruse et al. 2008). This model proposes that females require a greater load of susceptibility genes, in addition to other environmental and/or hormonal factors. Applied to our family, this model may explain why females show a less severe patellar anomaly compared to the affected males of the family.

From a genetic point of view, both a model of compound inheritance involving a combination of rare coding variants and common coding and noncoding variants in the *TBX4* gene and a model of digenic inheritance involving other genes have been hypothesized to account for the variable disease severity in affected patients (Karolak et al. 2023). According to this hypothesis, although our patients do not show pulmonary issues, we also investigated the presence of additional variants in the *TBX4* gene. This analysis allowed the identification of six additional variants, of which three (c.941C>T, c.1227C>T, c.*707G>T) are present in the proband only and inherited from the unaffected mother. The compound inheritance of these three variants added to the presence of the paternal c.735delT pathogenic variant may possibly account for the severe phenotype observed in the proband, but would not explain the severe phenotype in the father. However, since these additional variants are common in the general population (MAF > 5%) and are classified as benign according to the ACMG criteria, further studies are necessary to confirm their possible contribution to the pathomechanism of the most severe form of ICCPS.

Another effort to explain differences in disease severity was to explore the possibility of either a digenic condition or a dual molecular diagnosis, using the DIVA software (EnGenome). Of the 42 combinations that emerged from this analysis (Table S1), none was further investigated, since the function of the possible candidate genes for the digenic inheritance did not correlate with the phenotype.

In conclusion, ES analysis allowed us to reach a conclusive diagnosis of ICCPS in this family and to expand the spectrum of *TBX4* causative variants. The definition of the molecular diagnosis facilitates a more precise clinical management and follow-up of the patients from both the orthopedic and pulmonary point of view, as well as the possibility to define the precise recurrence risk and perform adequate preconceptional counseling.

Despite the additional analyses performed with the attempt to explain the differences in disease severity observed in the affected family members, the intrafamilial heterogeneity remains an open issue. Further studies, such as the analysis of intronic/regulatory regions, copy number variants (CNVs), epigenetic and gender-related factors, will be required to shed light onto the complexity of *TBX4* role in patellar development.

## Electronic supplementary material

Below is the link to the electronic supplementary material.

Supplementary Material 1

## Data Availability

The data that support the findings of this study are available from the corresponding author upon reasonable request.
