# Correction to: A novel frameshift *TBX4* variant in a family with ischio-coxo-podo-patellar syndrome and variable severity

**DOI:** 10.1007/s13258-024-01603-w

**Published:** 2025-01-27

**Authors:** Giada Moresco, Ornella Rondinone, Alessia Mauri, Rita Gorgoglione, Daniela Maria Grazia Graziani, Michal Dziuback, Monica Rosa Miozzo, Silvia Maria Sirchia, Luca Pietrogrande, Angela Peron, Laura Fontana

**Affiliations:** 1 Medical Genetics, Department of Health Sciences, Università degli Studi di Milano, Milan, Italy; 2 Research Laboratories Coordination Unit, Fondazione IRCCS Ca Granda Ospedale Maggiore Policlinico, Milan, Italy; 3 Medical Genetics, ASST Santi Paolo e Carlo, Milan, Italy; 4 Orthopedics and Traumatology Unit, ASST Santi Paolo e Carlo, Milan, Italy; 5 Orthopedics and Traumatology, Department of Health Sciences, Università degli Studi di Milano, Milan, Italy; 6 Present Address: Department of Biomedical and Clinical Sciences, Pediatric Clinical Research Center “Romeo ed Enrica Invernizzi”, University of Milan, Milan, Italy; 7 Present Address: Medical Genetics Laboratory, Fondazione IRCCS Ca’ Granda Ospedale Maggiore Policlinico, Milan, Italy; 8 Division of Medical Genetics, Meyer Children’s Hospital IRCCS, Florence, Italy; 9 Present Address: Department of Experimental and Clinical Biomedical Sciences “Mario Serio”, Università degli Studi di Firenze, Florence, Italy

**Correction to: Genes & Genomics** 10.1007/s13258-024-01589-5

The article “A novel frameshift TBX4 variant in a family with ischio-coxo-podo-patellar syndrome and variable severity”, written by Giada Moresco, Ornella Rondinone, Alessia Mauri, Rita Gorgoglione, Daniela Maria Grazia Graziani, Michal Dziuback, Monica Rosa Miozzo, Silvia Maria Sirchia, Luca Pietrogrande, Angela Peron, Laura Fontana, was originally published electronically on the publisher’s internet portal on 16 October 2024 without open access. With the author(s)’ decision to opt for Open Choice the copyright of the article changed on 1 December 2024 to © The Author(s) 2024 and the article is forthwith distributed under a Creative Commons Attribution 4.0 International License, which permits use, sharing, adaptation, distribution and reproduction in any medium or format, as long as you give appropriate credit to the original author(s) and the source, provide a link to the Creative Commons licence, and indicate if changes were made. The images or other third party material in this article are included in the article’s Creative Commons licence, unless indicated otherwise in a credit line to the material. If material is not included in the article’s Creative Commons licence and your intended use is not permitted by statutory regulation or exceeds the permitted use, you will need to obtain permission directly from the copyright holder. To view a copy of this licence, visit http://creativecommons.org/licenses/by/4.0. Open access funding enabled and organized by Projekt DEAL.

